# Human CD180 Transmits Signals via the PIM-1L Kinase

**DOI:** 10.1371/journal.pone.0142741

**Published:** 2015-11-10

**Authors:** Nicole Egli, Alexandra Zajonz, Matthew T. Burger, Tamas Schweighoffer

**Affiliations:** 1 Autoimmune, Transplantation, and Inflammatory Diseases Area, Novartis Institutes for Biomedical Research, Basel, Switzerland; 2 Global Discovery Chemistry, Novartis Institutes for Biomedical Research, Cambridge, MA, United States of America; IISER-TVM, INDIA

## Abstract

Toll-like receptors (TLRs) are important sensors of the innate immune system that recognize conserved structural motifs and activate cells via a downstream signaling cascade. The CD180/MD1 molecular complex is an unusual member of the TLR family, since it lacks the components that are normally required for signal transduction by other TLRs. Therefore the CD180/MD 1 complex has been considered of being incapable of independently initiating cellular signals. Using chemogenetic approaches we identified specifically the membrane bound long form of PIM-1 kinase, PIM-1L as the mediator of CD180-dependent signaling. A dominant negative isoform of PIM-1L, but not of other PIM kinases, inhibited signaling elicited by cross-linking of CD180, and this effect was phenocopied by PIM inhibitors. PIM-1L was directed to the cell membrane by its N-terminal extension, where it colocalized and physically associated with CD180. Triggering CD180 also induced increased phosphorylation of the anti-apoptotic protein BAD in a PIM kinase-dependent fashion. Also in primary human B cells, which are the main cells expressing CD180 in man, cross-linking of CD180 by monoclonal antibodies stimulated cell survival and proliferation that was abrogated by specific inhibitors. By associating with PIM-1L, CD180 can thus obtain autonomous signaling capabilities, and this complex is then channeling inflammatory signals into B cell survival programs. Pharmacological inhibition of PIM-1 should therefore provide novel therapeutic options in diseases that respond to innate immune stimulation with subsequently increased B cell activity, such as lupus erythematosus or myasthenia gravis.

## Introduction

Medicines used for targeted therapies achieved clinical success due to their very high efficacy and selectivity. Kinase inhibitors represent a particularly successful class of targeted drugs, and many of them can offer a nearly complete biochemical inhibition of the critical target protein, while effects on other non-target molecules are minimal or absent. Our recently discovered series of PIM kinase inhibitors are an eminent example of such selective and effective molecules [1]. Overexpression and upregulation of PIM kinases is often found in lymphomas and leukemias and in prostate cancer, and highly active and specific PIM inhibitors are predicted to improve the outcome of these malignancies.

Compound 5c of the series inhibits specifically all three members of the PIM kinases with picomolar biochemical and nanomolar cellular potency, is highly selective against other targets, and at the same time displays favorable cellular and physico-chemical properties [1]. We thus selected Compound 5c as a chemical probe that we used to reassess the role of PIM kinases beyond cancer, with a focus on a broad range of immune processes. Only little is known about the role of PIM kinases in inflammation: using genetically deficient mice, Pim-2 but not Pim-1 or -3, was shown previously to be required for IL-6 production upon stimulation of spleen cells with proinflammatory agents [2]. As part of an NFkB-driven loop, Pim-2 was also considered as a candidate that could phosphorylate and thus amplify the proinflammatory action of the Tpl2/Cot kinase in mice [3].

The family of toll-like receptors (TLRs) are transmembrane sensors of the innate immune system that elicit inflammatory responses when they recognize conserved patterns on microbial and endogenous targets. However, previous results indicated that Pim-2 was not an essential contributor to the pathway mediated at least by the toll-like receptor 4 (TLR-4), as LPS-induced TNFα and IL-10 were not decreased in the Pim-2 deficient cells [2]. Using our chemical probe, we tested functions of all conventional TLRs in cytokine release assays in human myeloid and B cell lines. Additionally we also extended the survey to CD180, which is a divergent member of the TLR family, being most related to TLR-4. CD180 and TLR-4 share a high degree of homology in their extracellular leucin rich repeat domains (LRRs), and while both of them form complexes with homologous smaller subunits (MD-2 and MD-1, respectively), the conformation of the ligand-bound CD180 dimers is profoundly different from that of TLR-4 [4]. In addition, CD180 lacks the intracellular TIR domain that would recruit MyD88, which in TLRs is generally required for downstream signaling [5]. With all these structural differences CD180 cannot signal like a conventional TLR molecule, yet productive signaling with the participation of CD180 or its murine counterpart Rp105 has been demonstrated in multiple settings [6–9]. In mice Rp105 can apparently cross-talk and compete for ligand binding with TLR4 [10], and also recruit elements from other established pathways for signaling [11,12]. However, the expression profile of TLR family members, including CD180, differs between mice and man. Particularly, in human peripheral B cells that are the highest expressors of CD180, TLR4 is essentially absent. Consequently, unlike in mouse models, it would be impossible for CD180 to borrow TLR4-associated components for its own use. Taken together, these findings indicated that there must be a novel autonomous CD180-directed signaling pathway. Here we show that such a CD180-dependent pathway indeed exists, and that CD180 achieves this by physically associating with PIM-1L, a special membrane-bound isoform of the PIM kinase family.

## Materials and Methods

### Inhibitors

CAS 491871-58-0 (Merck Millipore, Schaffhausen Switzerland) is an ATP-competitive PIM-1 inhibitor (biochemical IC50~50nM) selective over PIM-2 (IC50>20uM) and other kinases. CAS 587852-28-6 (Merck Millipore, Schaffhausen, Switzerland) is also an ATP-competitive inhibitor with a biochemical IC50~150nM against PIM-1 and IC50~20 nM against PIM-2. Discovery, synthesis and profiling of Compound 5c was described recently [1].

### Reagents

Full-length cDNAs of CD180, MD-1, PIM-1/2/3 were purchased from Origene Technologies. Kinase-dead mutants of PIM-1 (K67M) and PIM-2 (K61M) cDNAs were constructed by PCR using splice overlap extension. The coding sequences were in certain cases modified with in-frame tags or fluorescent proteins, and then recloned into plasmids that simultaneously direct the expression of mCD8a via an ECMV IRES element. The PIM-1 specific siRNA Hs_PIM1_1 (SI00040978), Hs_PIM1_6 (SI02758553), and PIM-2 specific siRNA Hs_PIM2_4 (SI00092869), Hs_PIM2_5 (SI02224201) were from Qiagen (Hilden Germany). Monoclonal anti-CD180 antibodies G28 and MHR23 were obtained from BD Pharmingen and from Abcam, respectively. The anti-GFP antibody was from eBiosciences and the polyclonal anti-PIM-1 antibody was obtained from Abcam.

## Cell culture

The established cell lines HEK293, THP-1, U937 and JeKo-1 were cultured in DMEM or RPMI1640 medium, supplemented with glutamine, antibiotics, and fetal calf serum. Stable lines were generated by electroporation of linearized plasmids followed by multiple rounds of sorting for surface mCd8a. Lines showing equal levels of mCd8a in FACS analysis were then used to ensure comparable levels of transgene expression. Human peripheral B cells were obtained by a negative B cell magnetic separation kit using AutoMacs (Miltenyi Biotech, Bergisch Gladbach, Germany).

### ELISA

Matched antibody pairs were used for ELISAs for measuring IL-8, TNFa, (DuoSets, R&D Systems), and IL-10 (ELISA Max Standard Set, BioLegend). Assays were performed according to manufacturer’s protocol.

### Proliferation Assay

Freshly isolated B cells were incubated for 48h with stimuli in the presence of Compound 5c. Tritium labeled thymidine was added to the cultures at a concentration of 5μCi/mL and incubated for another 24h. Cells were harvested on filtermats using the Tomtec Cell Harvester (Tomtec LifeSciences) and scintillation counts were measured using MicroBeta TriLux Scintillation Counter (Perkin Elmer).

### Immunoprecipitation and Western Blot

Cells were transiently transfected using TurboFect (Life Technologies), and then lysed using Cell Extraction Buffer (Life Technologies). Lysates were precleared with serum coated Protein G Sepharose (GE Healthcare) and then immunoprecipitated using either anti-APP (generated at NIBR) or anti-GFP antibodies (eBiosciences) and Protein G Sepharose. Precipitates were incubated at 56°C for 15minutes in LDS Sample Buffer (Life Technologies) and loaded on a 10% Bis Tris Mini Gel (Life Technologies). The gel was transferred using Transblot Turbo (BioRad). The membranes were blocked with Odyssey Blocking Buffer (LiCor) and stained with anti-PIM-1 (Abcam) and goat-anti-rabbit-IFR800CW (LiCor) antibodies. Membranes were processed using the Odyssey Infrared Imaging System (LiCor).

### Microscopy

Cells were transiently transfected using TurboFect (Life Technologies) on coverslips, fixed and counterstained with DAPI, mounted in Prolong Gold Antifade Reagent (Life Technologies), and analyzed on a Zeiss LSM 700 confocal laser scanning microscope.

### pBAD(Ser112) measurements

Where indicated, cells were pretreated with PIM inhibitors for 4h. Whole cell lysates were prepared by detergent lysis, and protein content was determined by the BCA method (Pierce, Rockford). Lysates were either directly blotted with pBAD S112 antibody (Cell Signaling Technology) [13], or phosphorylated BAD was quantitatively measured using equalized amounts of whole cell lysate proteins using a sandwich immunoassay with electrochemiluminescent read out (MesoScale Discovery, Gaithersburg, USA).

## Human samples

Peripheral blood mononuclear cells were collected under the Basel Tissue Donor Program (BTDP) study protocol from healthy volunteers after a written informed consent was obtained. BTDP was approved by the NIBR Bioethics Advisory Group as Institutional Review Board. Sample life-cycle management and record keeping, including planning, acquisition, collection, storage, release for use, anonymization and destruction, was performed in compliance with the Swiss Human Research Act (HRA; January 2014)

## Results and Discussion

### Cytokine release induced by TLR family members depends on intact PIM kinase activity

**Fig 1:**
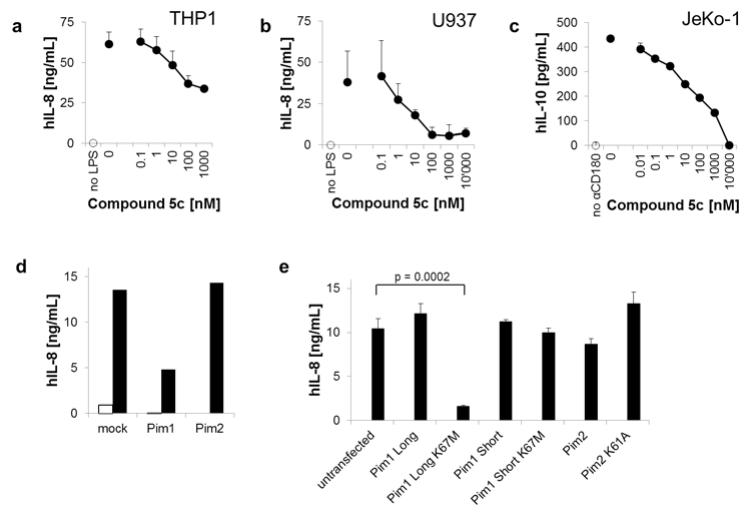
Cytokine release induced by TLR family members depends on intact PIM kinase activity. (a,b) THP-1 and U937 cells were treated with 10ug/ml LPS in the presence of logarithmic dilutions of Compound 5c. (c) JeKo-1 cells were treated with 1ug/ml anti-CD180 mAb G28 in the presence of logarithmic dilutions of Compound 5c. Representative results from more than five independent experiments covering different dose ranges are shown. (d) Various synthetic siRNA pairs were introduced into THP-1 cells, which were then left untreated (empty bars) or exposed to 10ug/ml LPS (filled bars). (e) Stable cell lines were generated by introducing plasmids encoding fully functional and kinase dead variants (K67M mutation for PIM-1, K61A mutation for PIM-2) into THP1 cells. Cells were then left either untreated (empty bars) or exposed to 10ug/ml LPS (filled bars). Representative results from at least three independent experiments are shown. In all assays overnight release of cytokines was measured by ELISA.

Functions of TLRs were first tested in cytokine release assays using human myeloid cell lines. These cells were confirmed to express PIM kinases as well as several TLR family members, and do react with vigorous release of IL-8 upon stimulation with cognate TLR ligands (S1 Fig). Compound 5c in a dose-dependent manner inhibited LPS-stimulated IL-8 release both from THP-1 cells (IC50 > 1um; Fig 1A) and from U937 cells (IC50~ 9 nM; Fig 1B). These results suggested that one or more PIM kinases had either an amplifying or a direct signaling role in the TLR-initiated cascades.

To evaluate CD180-dependent effects we tested various human cell lines that expressed both CD180 and PIM kinase(s). JeKo-1, a mantle cell lymphoma line, showed a dose- and time-dependent release of IL-10 upon treatment with anti-CD180 mAb, and was selected for further assays (S2 Fig). In this assay setup Compound 5c was completely inhibitory in a dose-dependent manner, suggesting that CD180 is capable of signaling in a PIM-dependent fashion (Fig 1C). Most importantly, inhibitory activity occurred with an IC50 = 27 nM, at a more than 100-fold lower dose than what is required for the biochemical inhibition of the closest irrelevant kinase target.

Contribution of PIM and its isoforms was then assessed with genetic tools. A PIM-1-specific siRNA, which also effectively decreased PIM-1 protein levels, significantly diminished release of IL-8 (Fig 1D). In contrast, blocking of PIM-2 did not suppress cytokine release. The gene structure of PIM-1 is, however, unusual: from the DNA-encoded gene a single mRNA is transcribed, from which then two PIM-1 protein variants are produced [14]. An alternative upstream CUG initiation codon instructs the production of a ~44 kDa PIM-1L isoform besides the shorter 34 kDa form, which both contain the kinase domain. Since the two isoforms originate from the same message, both were suppressed by siRNA treatment. We therfore constructed dominant negative (DN) acting kinase dead mutants of each isoform, which were expressed separately in the cells. Surprisingly, we found that only the DN variant of the long (44kDa) form of PIM-1 suppressed the release of IL-8 (Fig 1E).

### CD180 colocalizes and associates with Pim-1L

**Fig 2:**
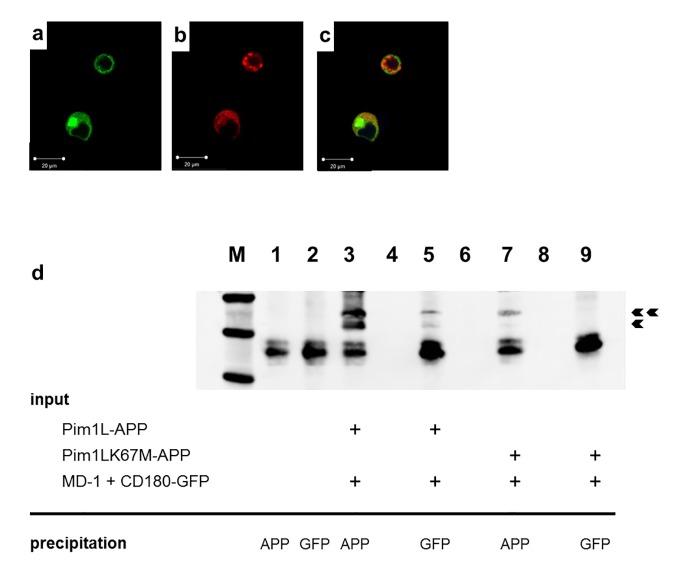
CD180 colocalizes and associates with Pim-1L. (a-c) HEK cells were grown on a coverslip, and were cotransfected with plasmids encoding fluorescently labelled CD180 (green, panel a) and PIM-1L (red, panel b). Confocal fluorescence microscopy indicated colocalization of CD180 and PIM-1L preferentially in the plasma membrane, and also in some subcellular compartments. (d) Coimmunoprecipitation of transfected cells. Various combinations of plasmids encoding APP-tagged PIM-1L and EGFP-tagged CD180 were introduced into HEK cells as indicated by the (+) marks. Equal amounts of cellular proteins were immunoprecipitated with the respective anti-tag antibodies as indicated. The total precipitate was then resolved on a denaturing gel, and blotted with an anti-PIM-1 antibody. Double and single arrowheads indicate the long and the short forms of the PIM-1 protein, respectively. Representative results from three independent experiments are shown.

The long form PIM-1L contains an extra proline-rich motif at the N-terminus that anchors this variant to membranes and also allows intermolecular associations. Indeed, PIM-1 was not only detected at the cytosolic membranes and in the nucleus, but also at the surface membrane of human and murine cancer cells [15–17]. We thus reasoned that PIM-1L may directly interact in the cell membrane with CD180, and that this association can then transmit signals which originate from the extracellular face of CD180. Both molecules were fused to fluorescent proteins, green and red respectively, on their C-terminal end, and transfected together with an unlabeled MD-1 construct into HEK cells. Using confocal microscopy we found that both proteins are mainly localized to membranes, primarily to the plasma membrane (Fig 2A and 2B). An overlay of the individual channels indicated a high degree of colocalization of the labelled proteins (Fig 2C).

Additionally, coprecipitation experiments were performed with tagged constructs to prove a physically stable association of PIM-1L and CD180 (Fig 2D). The PIM-1L vector directed the expression of both the long and the short form of the protein (double and single arrowheads, respectively) which were both tagged with a C-terminal APP tag, and also recovered in the anti-APP-tag precipitate (lane 3). When the immunoprecipitation was performed with an anti-GFP antibody directed against the CD180 fusion protein, PIM-1 proteins were still recovered, with a preference of the PIM-1L form over the short variant (lane 5). A tagged, kinase-dead DN PIM-1L variant was tested similarly. While possibly due to structural reasons the overall expression of this construct was weaker in general (lane 7), no association with CD180 was detectable any more (lane 9).

Colocalization and coimmunoprecipitation experiments indicated that a physically close interaction does exist between CD180 and PIM-1L. It remains to be determined whether the transmembrane or intracellular portions of both molecules can interact directly without further help, or if other molecules, such as Hsp90, BCRP, or Etk, which were previously shown to interact with PIM-1, are also contributing to the formation of a signaling complex [16,18]. That only the intact PIM-1L but not the kinase deficient, dominant negatively acting K67M, was interacting with CD180, suggested that the kinase function may be required either to maintain conformational integrity of the PIM-1L protein, or directly for stabilizing its association with CD180. A similar observation was made by Kim et al., who also found that the K67M variant was not a substrate of an LMP1-induced intracellular translocation, in contrast to the unmodified PIM-1 [19].

### CD180 stimulation induces PIM-dependent phosphorylation and proliferation

**Fig 3:**
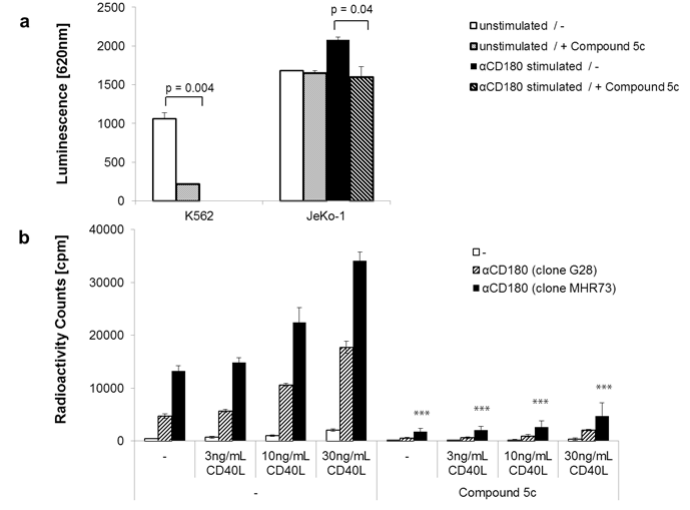
CD180 stimulation induces PIM-dependent phosphorylation and proliferation. (a) Phosphorylation of BAD(Ser112) was determined from equal amounts of whole cell lysates using a MesoScale immunoassay. JeKo-1 cells were tested without (empty bars) or with stimulation by CD180-specific antibodies (filled bars); and in parallel, the same was done in the presence of 1uM Compound 5c (thin and thick stripes, respectively). K562 cells, either untreated (empty bars) or also treated with 5c (thin stripes) were used for comparison. Representative results from three independent experiments are shown. Significant differences between tested groups are indicated by brackets and p-values. (b) Primary human B cells purified by negative magnetic selection were stimulated by adding CD180-specific antibodies (striped bars = clone G28; filled bars = clone MHR23). A multimeric CD40L was included in increasing concentrations (0, 3ng/ml, 10ng/ml, and 30ng/ml), with or without the presence of Compound 5c (1uM). Proliferation was measured by 3H-thymidine incorporation after four days. Stars (***) indicated significant differences p<0.001 between pairs of treated vs. untreated samples. Results from one donor out of four tested with similar results shown.

A hallmark target of PIM kinases is the Ser112 residue of the anti-apoptotic protein BAD, whose phosphorylation can be readily quantified by a site-specific immunoassay. In exponentially growing K562 cells, this modification is spontaneously and almost exclusively performed by PIM-1; accordingly, Compound 5c applied at 1uM (corresponding to the average IC90 level of cellular assays) suppressed the pBAD signal close to background in K562 cells (Fig 3A; also S3 Fig). In contrast, BAD is a target of multiple kinases in JeKo-1 cells, which therefore display a high background of PIM independent, spontaneously phosphorylated BAD. Nevertheless, signals from CD180 did additionally and significantly increase pBAD levels, and this CD180-induced part was then completely inhibited to background by Compound 5c.

We also examined whether the CD180-initiated, PIM-dependent signaling pathway was functional in freshly isolated human peripheral B cells. Already low concentrations of two independent anti-CD180 antibody clones induced proliferation on their own, without the need of further crosslinking (Fig 3B). As expected, proliferation was increasingly potentiated by the inclusion of increasing amounts of CD40L, since CD40 further up-regulates PIM-1 mRNA expression [20]. Compound 5c inhibited proliferation in all cases close to background levels. These data indicate that the CD180/PIM pathway is functional in human B cells, and suggest that PIM inhibitors could be applied to therapeutically modulate B cell functions.

## Conclusions

Our data show that CD180 is capable of initiating signals independently from the known TLR-dependent pathways via the PIM-1L kinase isoform. In humans, B cells express the highest levels of CD180, while they are essentially devoid of TLR-4 and several other surface TLR family members. Thus the CD180/PIM-1L pathway must be especially important for human B cell functions, and contribute to signaling leading to cell activation, induction and affinity maturation of antigen-specific IgG, and immunological memory [9,21,22]. CD180 can also provide powerful expansion and survival signals for B-CLL leukemic cells [23]. In addition, the CD180/MD-1 complex was also implicated in chronic tissue inflammation, particularly in the adipose tissue [24]. Polymorphism in the MD-1 (LY86) locus was found to be associated with obesity and body fat distribution in certain populations in unbiased genome wide studies [25].

Combining published data with our findings enables us to speculate that the CD180 / PIM-1L axis, functioning as an anti-apoptotic pathway, would be also a major contributor to radiation resistance. On the one end, CD180 (Rp105) cross-linking protected B cells from irradiation-induced apoptotic cell death [7]. On the other end, PIM-1 was also shown to be required for radioresistance in murine cancer models [26]. In humans, PIM-1 mRNA was upregulated in multiple human solid cancers, and moderate to high expression of PIM-1 correlated with poor clinical response to radiation therapy [27].

In conclusion, these data suggest that the CD180 / PIM1L axis is important for transmitting inflammatory and survival signals, and consequently, its pharmacological inhibition may provide novel therapies in autoimmune-inflammatory diseases. Because animals lacking all PIM genes are viable with a mild phenotype, one could expect that a therapeutic approach targeting either PIM-1 selectively, or broadly all PIM family members, like Compound 5c does, could be applied with acceptable side effects [28].

## Supporting Information

S1 Fig IL-8 release induced by TLR ligands is dependent on Pim kinases. THP-1 cells were stimulated with various ligands specific for different TLRs. The stimulation was performed in the presence of the inhibitor Compound 5c (10uM). Overnight release of IL-8 was determined by ELISA.
(PDF)

S2 Fig Anti-CD180 mAb treatment induces IL-10 release in JeKo-1 cells. (a) Various B cells lines were cocultured with the anti-CD180 mAb G28 (1ug/mL), and release of IL-10 at the indicated timepoints was determined by ELISA. BJAB cells (diamonds with dashed line) spontaneously secrete IL-6 and IL-10. JeKo-1 cells (triangles with solid line) express both CD180 and Pim-1 at medium-high levels, and produce IL-10 upon CD180 stimulation. SUDHL4 cells (X marks with dotted line) are deficient in IL-10 release. (b) In JeKo-1 cells CD180 stimulation-induced IL-10 production is suppressed by chemical inhibitors of Pim. CAS 491871-58-0, and CAS 587852-28-6 were applied at 20uM, Compound 5c was used here at 10uM. Differences in the grade of inhibition reflect the potency of the inhibitors.
(PDF)

S3 Fig PIM-dependent phosphorylation of BAD inhibited by Compound 5c. K562 cells were pretreated with PIM inhibitors for 4h, with or without concomitant activation using PMA/ionomycin. Whole cell lysates were prepared by detergent lysis, and protein content was determined by the BCA method (Pierce, Rockford). Lysates were either blotted with P-BAD S112 antibody. Compound 5c inhibits steady-state phosphorylation of BAD in K562 cells; this is overridden by the broadly activating PMA/ionomycin treatment. HEL cells with constitutively high, PIM-independent pBAD levels are shown for comparison.
(PDF)
