# Supplementary figures and images for: Human CD180 Transmits Signals via the PIM-1L Kinase
Source: PLoS One. 2015 Nov 10;10(11):e0142741. doi: 10.1371/journal.pone.0142741 (PMC4640547; doi:10.1371/journal.pone.0142741)

Supplementary Figure S1

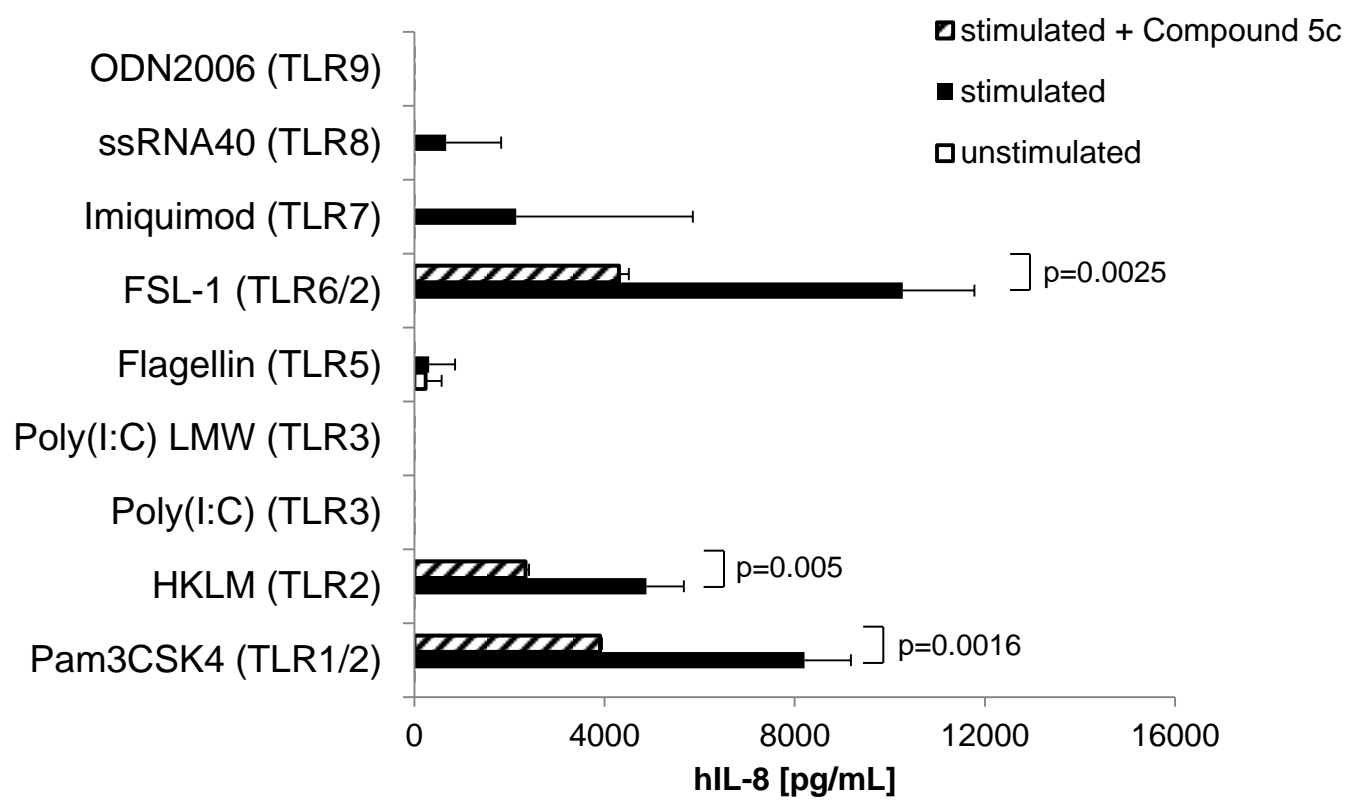

Supplement: S1 Fig — THP-1 cells were stimulated with various ligands specific for different TLRs. The stimulation was performed in the presence of the inhibitor Compound 5c (10uM). Overnight release of IL-8 was determined by ELISA. (PDF) [file pone.0142741.s001.pdf]

Supplementary Figure S2

**a**

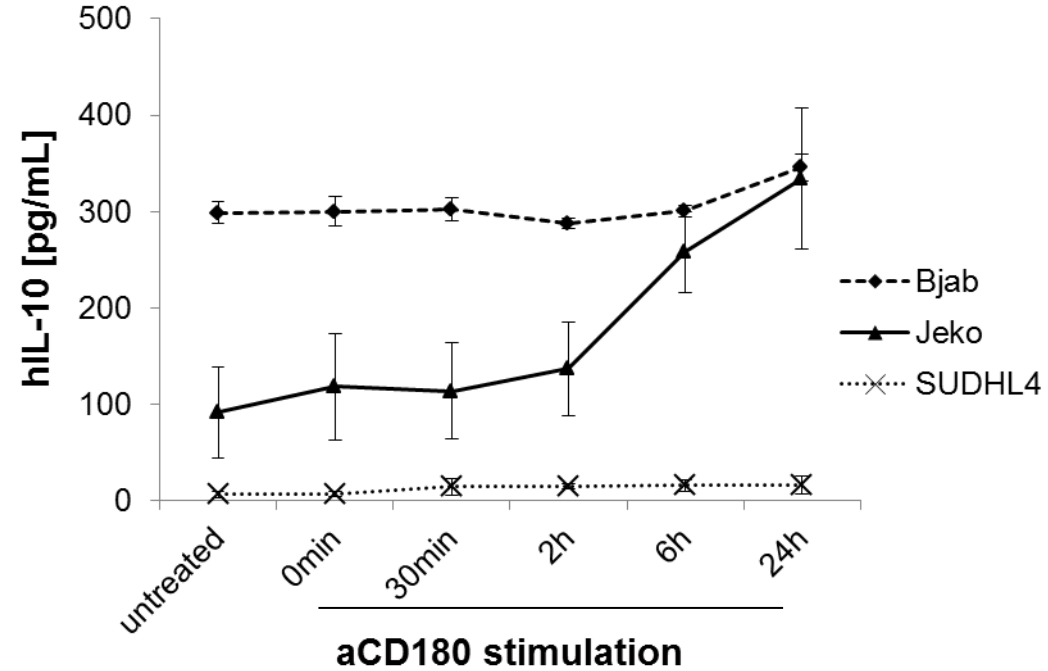

**b**

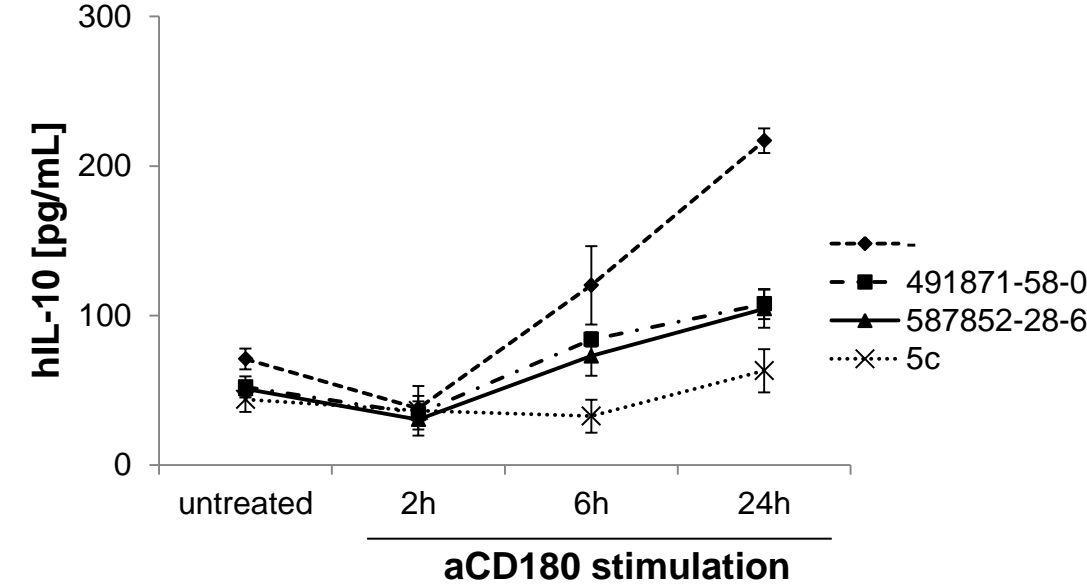

Supplement: S2 Fig — (a) Various B cells lines were cocultured with the anti-CD180 mAb G28 (1ug/mL), and release of IL-10 at the indicated timepoints was determined by ELISA. BJAB cells (diamonds with dashed line) spontaneously secrete IL-6 and IL-10. JeKo-1 cells (triangles with solid line) express both CD180 and Pim-1 at medium-high levels, and produce IL-10 upon CD180 stimulation. SUDHL4 cells (X marks with dotted line) are deficient in IL-10 release. (b) In JeKo-1 cells CD180 stimulation-induced IL-10 production is suppressed by chemical inhibitors of Pim. CAS 491871-58-0, and CAS 587852-28-6 were applied at 20uM, Compound 5c was used here at 10uM. Differences in the grade of inhibition reflect the potency of the inhibitors. (PDF) [file pone.0142741.s002.pdf]

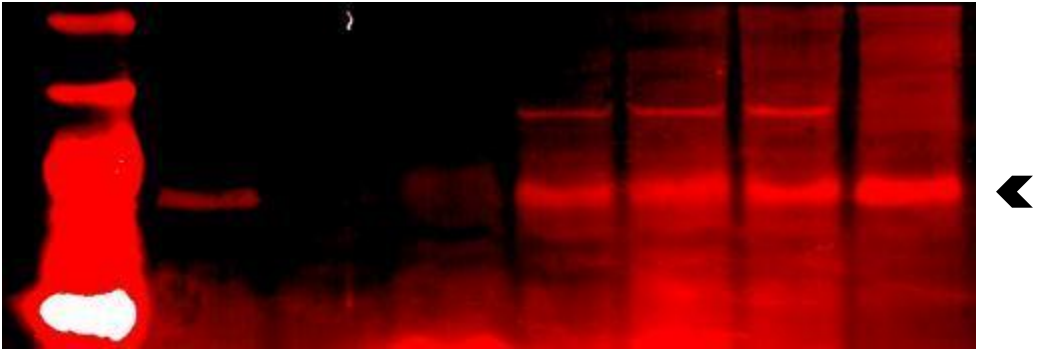

|                 |   |      |     |   |      |     |   |
|-----------------|---|------|-----|---|------|-----|---|
| K562 cells      | + | +    | +   | + | +    | +   |   |
| HEL cells       |   |      |     |   |      |     | + |
| PMA / ionomycin |   |      |     | + | +    | +   |   |
| Compound 5c     | - | 10uM | 1uM | - | 10uM | 1uM | - |

Supplement: S3 Fig — K562 cells were pretreated with PIM inhibitors for 4h, with or without concomitant activation using PMA/ionomycin. Whole cell lysates were prepared by detergent lysis, and protein content was determined by the BCA method (Pierce, Rockford). Lysates were either blotted with P-BAD S112 antibody. Compound 5c inhibits steady-state phosphorylation of BAD in K562 cells; this is overridden by the broadly activating PMA/ionomycin treatment. HEL cells with constitutively high, PIM-independent pBAD levels are shown for comparison. (PDF) [file pone.0142741.s003.pdf]
